# The Impact of the Wenchuan Earthquake on Birth Outcomes

**DOI:** 10.1371/journal.pone.0008200

**Published:** 2009-12-07

**Authors:** Cong E. Tan, Hong Jun Li, Xian Geng Zhang, Hui Zhang, Pei Yu Han, Qu An, Wei Jun Ding, Mi Qu Wang

**Affiliations:** 1 Institute of Basic Medicine, Chengdu University of Traditional Chinese Medicine, Chengdu, China; 2 Shaanxi University of Chinese Medicine, Xi'An, China; Aga Khan University, Pakistan

## Abstract

**Background:**

Earthquakes and other catastrophic events frequently occurring worldwide can be considered as outliers and cause a growing and urgent need to improve our understanding of the negative effects imposed by such disasters. Earthquakes can intensively impact the birth outcomes upon psychological and morphological development of the unborn children, albeit detailed characteristics remain obscure.

**Methods and Findings:**

We utilized the birth records at Du Jiang Yan and Peng Zhou counties to investigate the birth outcomes as a consequence of a major earthquake occurred in Wenchuan, China on May 12, 2008. Totally 13,003 of neonates were recorded, with 6638 and 6365 for pre- and post- earthquake, respectively. Significant low birthweight, high ratio of low birthweight, and low Apgar scores of post-earthquake group were observed. In contrast, the sex ratio at birth, birth length and length of gestation did not show statistical differences. The overall ratio of birth-defect in the post-earthquake (1.18%) is statistically high than that of pre-earthquake (0.99%), especially for those in the first trimester on earthquake day (1.47%). The birth-defect spectrum was dramatically altered after earthquake, with the markedly increased occurrences of ear malformations. The ratio of preterm birth post-earthquake (7.41%) is significant increased than that of pre-earthquake (5.63%). For the birth outcomes of twins, significant differences of the ratio of twins, birth weight, ratio of low birthweight and birth-defect rate were observed after earthquake.

**Conclusion:**

A hospital-based study of birth outcomes impacted by the Wenchuan earthquake shows that the earthquake was associated with significant effects on birth outcomes, indicating it is a major monitor for long-term pregnant outcomes.

## Introduction

On May 12, 2008, a catastrophic earthquake measuring 8.0 on the Richter scale hit Wenchuan, in southwestern China, leading to 69,227 deaths and 374,643 injuries, with 17,923 listed as missing [1]. This devastating earthquake struck the eastern edge of the Tibetan plateau, collapsing buildings and killing thousands in major cities aligned along the western Sichuan basin in China [2]. It destroyed around 70% of houses and at least 85% of local public buildings, and shook the whole nation [1]–[3]. Consequently, Du Jiang Yan, Peng Zhou and other counties surrounding the earthquake center, Wenchuan, were simultaneously trapped. More than 40 million citizens were impacted, including about 270,000 pregnant women and 12 million women of child-bearing age [4].

Major earthquakes and other catastrophic events can be considered as outliers and result from mechanisms involving amplifying critical cascades [5]. Unfortunately, societies are subjected to various disasters, ranging from natural catastrophes, such as volcanic eruptions, hurricanes and tornadoes, landslides, avalanches, to the failure of engineering structures, social unrest, national and global economic downturns, regional power blackouts, diseases and epidemics, etc [2]–[5]. Hence, there is a growing and urgent need to grasp the intermittent dynamics of disasters in order to improve our understanding of the negative effects imposed by such occurrences.

Birth outcomes could be intensively impacted by all kinds of environmental calamities. The effects of a major earthquake can significantly impact the psychological and intellectual development of fetus [6]. Saadat [7] reported that a severe earthquake in Iran decreased the sex ratio at birth 6–12 months later as a result of psychological tensions and stress associated with the earthquake. Catalano et al.[8] pointed out that the Sept. 11, 2001 terrorist attack in New York City reduced conception of males and increased fetal deaths among males. Chang and colleagues [9] observed much high proportions of minor psychiatric morbidity (29.2%) and low birth weight neonates (7.8%) in a group of women who were pregnant during or immediately after a major earthquake. Based on the established rule that reproductive health is primary to societal growth and evolution, analysis of the effects of natural or man-made disasters upon pregnancies is a subject worthy of investigation.

In this study, we evaluated the birth outcomes before and after the Wenchuan earthquake. Here we report profound effects of the earthquake upon birth-defect, birth weight and sex ratio at birth and other primary characteristics. Our data suggest that rare catastrophic events are often a major monitor for long-term reproductive health and pregnant outcome in the affected population.

## Methods

### Objectives

This work was conducted according to the principles expressed in the Declaration of Helsinki, and approved by the Ethics Board, Chengdu University of Traditional Chinese Medicine. We employed exactly two year's data of birth redords. Those collected from May 12, 2007 through May 11, 2008 were put into a pre-earthquake group, while samples collected from May 12, 2008 through May 11, 2009 were included as a post-earthquake group. All participants signed informed consent before investigation. All parents or guardians of the neonates gave informed consent to publication of their case details in a style of anonymity. We began the observation on July 25, 2008. Of those delieved after July 25, 2008, their parents consented at the time of their usual hospital care during the birth. While for those born on July 25, 2008 or before, the informed consents were signed within the homes of their parents or guardians prior to the utilization of their medical records. Although some birth records were retrospectively collected, there has no substantive discrepancy on the collected data, because they are all objective and quantitive data recoded in the medical records of all neonates [7]–[9].

Two representative areas, Du Jiang Yan and Peng Zhou counties, were chosen for the research. Both of them are located less than 20 kilometers from the earthquake center, Yin Xiu Town in Wenchuan County, and were severely damaged. Samples were collected from every local hospital.

### Data Process

Every case of parturition occurring in the observed counties during survey period was included in the analysis. Major data for pregnant outcome, including age of pregnant women, birth length, birth weight and length of pregnancy, were evaluated by two independent researcher groups. Data criteria were as follows:

1. Low-birth weight neonates: defined as birth weight <or = 2500 gram.
2. Preterm infants: defined as length of gestation <or  = 36 weeks.
3. Birth defects: referred to concepts defined in gynecological textbooks. As the first trimester is a key period for organ formation, we further classified these into three groups, e.g. first trimester pregnancy on earthquake day, second and third trimester pregnancy on earthquake day, and post-earthquake pregnancy.
4. Apgar score: a standard evaluation for neonates, performed within one minute or five minutes after birth [6], [9]. We employed 1-Min and 5-Min Apgar scores in the study. This appraisal system includes five physical signs: skin color, heart beat, breath, muscle tension/movement, and reflexes. The healthy neonates are scored as 8–10 points; for slight distress, 4–7 points; while for severe distress, <4 points.

### Statistical Methods

All data were analyzed using SPSS version 15.0 (SPSS Inc., Chicago, IL). We generated descriptive statistics and bivariate associations for all variables and examined them for distributional normality. Numbers fit to normal distribution were analyzed by two-tailed t test and/or chi 2 test. For data that did not meet the distributional normality, a Mann-Whitney U test was applied. The significant differences were defined as P value less than 0.05.

## Results

A total of 13,003 of neonates were recorded in the local hospitals of Du Jiang Yan and Peng Zhou, two counties severely devastated by the Wenchuan earthquake. For general maternal index, no significant differences were observed between pre-earquake group and post-earthquake group (Table 1). The pre- and post- earthquake neonates were 6,638 and 6,365, respectively (Table 2). Which indicate that there was a 4.29% birthrate decrease after the earthquake. The ratio of preterm birth post-earthquake (7.41%) is significant increased than that of pre-earthquake (5.63%). We also observed statistical lower birthweight and Apgar scores, higher ratio of low birth weight and the rate of premature births, of the post-earthquake group compared to those of the pre-earthquake group. In contrast, no significant differences were observed in the sex ratio at birth, birth length, length of gestation, or general characteristics of pregnant women (Table 2).

**Table 1:** General characteristics of maternity participating in the study.

Category	Pre-earthquake group	Post-earthquake group	p-Value
Age (years), Mean±SD	24.29±4.15	24.96±4.99	>0.05
Weight (kg), Mean±SD	50.87±5.50	51.05±3.99	>0.05
Spontaneous birth (%)	70.86	73.67	>0.05
Education level (%)			
Primary school or less	32.62	31.19	>0.05
Junior middle school	35.36	37.55	>0.05
High school	24.70	24.25	>0.05
College or higher	7.32	6.71	>0.05
Multipara (%)	25.72	26.73	>0.05
Primipara (%)	74.28	73.21	>0.05

No statistic differences were observed between pre- and post- earthquake group. The p-Values were obtained by t test or X^2^ test. All data were collected at the delivery day. The significant differences were defined as P value less than 0.05.

**Table 2:** Birth outcomes of neonates delived pre- and post- Wenchuan earthquake.

Category	Pre-earthquake group	Post-earthquake group	p-Value
NO. of overall births	6638	6365	>0.05
Gestation length (wks), Mean±SD	39.71±2.28	39.06±2.41	>0.05
Birthweight (g), Mean±SD	3417.82±473.90	3251.37±491.88	0.05
Ratio of low birthweight (%)	3.72	5.01	<0.01
Ratio of preterm delivery (%)	5.63	7.41	<0.01
Birth length (cm), Mean±SD	49.92±1.14	48.97±1.15	>0.05
Ratio of male infants (%)	51.85	51.02	>0.05
1-Min Apgar score, Mean±SD	9.08±1.75	8.30±1.29	<0.01
5-Min Apgar score, Mean±SD	9.42±1.06	8.95±1.42	<0.05

During our survey periods, 141 infants with birth-defect were observed. Compared with healthy neonates (as listed in Table 2), the average birth weight of infants with birth-defects decreased but did not reach statistical significance; yet the ratio of low birthweight neonates with defects was statistically significant (Table 3). For birth-defect infants, the male ratio at birth was quite higher than that of healthy ones, especially those in the post-earthquake group. Results of Apgar scoring of birth-defect infants in both pre- and post- earthquake groups were markedly lower than that of healthy controls, with an even lower score observed in the post-earthquake group.

**Table 3:** Records of birth-defect neonates delived pre- and post- Wenchuan earthquake.

Category	Pre-earthquake group	Post-earthquake group	p-Value
Birth-defect neonates, N (%)	66 (0.99)	75 (1.18)	<0.05
Materal age (years); Mean±SD	26.02±3.21	25.79±3.35	>0.05
Length of gestation (wks); mean±SD	39.14±2.55	38.92±4.58	>0.05
Birth weight (g); Mean±SD	3214.51±500.57	3299.21±395.43	>0.05
Ratio of low birthweight neonates (%)	8.52	10.78	<0.01
Birth length (cm); Mean±SD	49.21±2.97	49.42±3.59	>0.05
Ratio of male infants (%)	59.67	68.83	<0.01
1-Min Apgar score; Mean±SD	8.25±1.69	7.46±1.04	<0.05
5-Min Apgar score; Mean±SD	8.92±2.06	8.42±1.33	<0.05

The overall ratio of birth-defect in the post-earthquake group was 1.18%, statistically higher than that of the pre-earthquake group (0.99%). Importantly, neonates delivered from Dec. 12, 2008 through Feb. 11, 2009, who were in the first trimester on the day of the earthquake, demonstrated a much higher ratio of birth-defects than either pre-earthquake group or other post-earthquake groups (Table 4).

**Table 4:** Birth defects recorded around the Wenchuan earthquake.

Gestation period on earthquake day	survey period	Total infants, N	Birth-defect infants, N (%)
**Pre-earthquake**	2007.05.12–2008.05.11	6638	66 (0.98)
**1^st^ trimester**	2008.12.12–2009.02.11	1155	17 (1.47) **$$
**2^nd^,3^rd^ trimester**	2008.05.12–2008.12.11	3886	44 (1.13) *
**Post-earthquake**	2009.02.12–2009.05.11	1324	14 (1.06)

Compared with pre-earthquake group: *P<0.05, **P<0.01;

Compared with other post-earthquake groups: $$P<0.01.

We further subdivided the categories of birth defects (Table 5). The results showed that cleft lip/palate, polydactylia, microtia/anotia were the most frequent occurring birth defects in our survey region. However, birth defects related to ears in the post-earthquake group were dramatically increased when compared with the pre-earthquake group. Of particular interest was the observation that in the patients who were in their first trimester on the day of the earthquake, more than half of their birth defects involved the ears.

**Table 5:** Top five birth defects occurred pre- and post- Wenchuan earthquake.

Group	Name of birth-defect	Frequency; N(%)	Occurrence per 10,000 births
Pre-earthquake	Cleft lip/ palate	17 (26.69)	26.7
	polydactylia	10(14.97)	15.0
	Microtia/notia	9(13.57)	13.6
	ankylodactylia	5(7.84)	7.8
	Equinovarus	4(6.02)	6.0
Post-earthquake	Microtia/notia	10(15.71)	15.7
	Cleft lip/ palate	7(10.99)	11.0
	polydactylia	6(9.43)	9.4
	Equinovarus	6(9.43)	9.4
	undescended testis	4(6.35)	6.4
1^st^ trimester on earthquake day	Microtia/ anotia	7(41.18)	60.6
	other auris deformity	3(17.65)	25.9
	polydactylia	2(11.76)	17.3
	Limb shortening	2(11.76)	17.3
	/	/	/

Table 6 shows that significant differences of four indices, i.e. the ratio of twins, birth weight, ratio of low birthweight and ratio of birth-defects, were observed in twins delivered before and after the earthquake. Which indicate in a unique aspect the significant impact of the earthquake upon the basic index of birth outcome. Although no statistical differences were observed in two indices (gestational time and birth length), there was a trend to differences when compared with single births.

**Table 6:** Twins born pre- and post- Wenchuan earthquake.

Category	Pre-earthquake group	Post-earthquake group	p-Value
NO. of twins	35	46	<0.05
Ratio of twins (‰)	5.27	7.23	<0.01
Gestation weeks, Mean±SD	38.45±1.02	36.74±2.72	>0.05
Birth length (cm), Mean±SD	47.12±2.01	46.38±3.33	>0.05
Birth weight (g), Mean±SD	2591.45±225.72	2412.76±295.43	<0.05
Ratio of low-birth weight neonates (%)	42.65	55.65	<0.01
Ratio of birth-defect twins (%)	3.01	9.43	<0.01

## Discussion

The Wenchuan earthquake was one of the most serious natural disasters in Chinese history. More than 250,000 square kilometers of Chinese land was ruined, 89,150 persons were killed or reported as missing [1]–[3]. A sustained occurrence of more than 20,000 aftershocks have been ceaselessly stressing local earthquake refugees, about 125,000 families are still live in temporary houses due to remaining earthquake debris. Although the earthquake was occurred in an under-populated area, about 27,000 neonates have been born in the affected regions [4].

It is established that antenatal stress is associated with various adverse birth outcomes in humans and other animals [10]. Therefore, the effect of natural disasters such as the Wenchuan earthquake on pregnant women is important and worthy of analysis. Although several publications have documented maternal vulnerability in earthquakes, few studies have completely examined the effects of earthquake on pregnant women and their neonatal outcomes [11]. Several prior studies suggest that prenatal stress caused by disasters may result in low birth weight [9], preterm delivery [12], skewed sex ratios [8], [13], and retardation of fetal brain development [10], [14]. Therefore, in this study we investigate the relationship between a major earthquake and birth outcomes, in order to further understand the pathobiological processes of such disasters, and define the types of support for pregnant women that should be provided in order to increase the likelihood of a normal pregnancy.

The most notable result of the present study was the significantly high ratio of birth-defects connected with the Wenchuan earthquake. There is little data regarding our observation that has been previously reported in the medical literature. Montenegro et al. [15] found that earthquake-induced stress significantly increased the rate of facial clefts. Castilla and Orioli [16] suggested that both psychological stress and the air pollution from the dust and gases emitted during the earthquake induced adverse birth outcomes. Our data indicates that the Wenchuan earthquake markedly increased the birth-defect rate, with 11.78 per 1,000 births in contrast to 9.94 per 1,000 births in previous years.

Interestingly, neonates who were in the first trimester on the day of the earthquake displayed a statistically significantly higher birth-defect ratio when compared with other post-earthquake groups. Consistent with this observation is that several reports have shown that pregnancy is more susceptible to stress effects in the first trimester than during the second and third trimesters [5], [6], [17]. The first trimester is the period for the beginning of morphogenesis and the most biologically vulnerable stage for the effects of diverse environmental factors. Accordingly, it is one of the key periods for birth-defect as well. Constant air pollution and water pollution resulting from the earthquake itself, and thousands of aftershocks with the associated stress caused by these events, might be important determining factors that contribute to the abnormal high ratio of birth-defect about the first trimester fetus and their development in post-earthquake niches. It has been hypothesized that as pregnancy advances, women become increasingly resistant to the adverse effects of stress, and so early stress would have more profound effects than later stress [14], [18]. Our results strongly suggest that first trimester is the most sensitive period that would be affected by the prolonged negative environmental factors resulting from the earthquake.

Another unexpected finding is the apparently changed spectrum of birth defects seen associated with the Wenchuan earthquake (Table 5). The average incidence of microtia and anotia in China was 1.40 per 10,000 during 1989–1992 [19]. However, the incidences of microtia/anotia were 15.7 and 60.6, for overall post-earthquake and first trimester group, respectively (Table 5). Ear malformations were significantly increased after Wenchuan earthquake, especially for those of first trimester on the day of the earthquake. In comparison, the ratio of cleft lip and/or palate didn't statistically increased in post-earthquake neonates, in contrast to other studies [14], [20]. These patterns of birth defects following the Wenchuan earthquake are not easily explained, and points to the need for more data on the role of environmental stress and fetal development.

A main finding in this study is that the average post-earthquake birthweight was statistically decreased. Lower birth weight is usually associated with increased fetal mortality, neonatal mortality, infant mortality and increased susceptibility to stress in adulthood [14]. Our results indicate that both the average birth weight and the low birthweight rate were significantly altered in the post earthquake group as compared to the pre-earthquake group (Table 2). Additionally, the statistically significant increased preterm delivery rate after the earthquake is interrelated with the abnormally high ratio of low birthweight neonates (Table 2). The etiology of both low birth weight and preterm delivery is complex and no single factor explains most of the variance in the rates of these birth outcomes. Instead, many biological, behavioral and social factors may impact these outcomes. Notwithstanding, this data can offer some medical practitioners important information regarding the type of care necessary for pregnant women immediately after a natural or man-made disaster.

It is worth noting that the ratio of low birthweight (5.01%) observed after the Wenchuan earthquake is lower than that of previously reported, such as that recorded after the Taiwan major earthquake (7.8%) [9]. Even if the factors influencing low birthweight are not well understood, this decrease in vulnerability may imply increasing protection of the mother and fetus from additional adverse influences after the Wenchuan earthquake [14]. Furthermore, additional birth outcomes, such as birth length and length of gestation, were not statistical different before and after the Wenchuan earthquake further suggesting that favorable care of pregnant women did ameliorate at least some aspects of the birth outcomes.

We do not observe the skewed sex ratio at birth within the survey period. Several publications reported that the human secondary sex ratio falls in populations subjected to exogenous stressors such as earthquakes or political and social upheavals [7], [8], [13]. Explanations of the association include reduced conception of males and increased fetal deaths among males [7], [8]. Simultaneously, a reduction in fertility was also observed in these reports. We investigated the shrinkage of fertility after the Wenchuan earthquake, taking into consideration a possible change of the female population of reproductive age. However, we did not observe any marked declination of sex ratio at birth after the Wenchuan earthquake. Fukuda et al. [13] found a sex ratio decline appeared in about 280 days following the earthquake, thus indicating the need for a long term analysis of the consequences of the Wenchaun earthquake.

The birth outcomes of twins suffering from prenatal disaster, Wenchuan earthquake, is of interest. Our results indicate that earthquake could increase the ratio of twins, and extensively impair the birth outcomes (Table 6). Furthermore, the overall results indicate that twins were more vulnerable to adverse environmental factors during fetus development than that of single births. Quite few reports have considered the impacts of natural disasters upon the outcomes of twins. Some prior studies employed twins as useful subjects to probe the effects of disasters on fetal development, but had not considered the birth outcomes [17], [18]. Bornstein et al. [12] reviewed that twin gestation is associated with a high risk for different subtypes of preterm birth; yet they ignored the impact of earthquake upon the birth outcomes of twins. Wadhawan et al. [11] found that twin gestation in extremely low birthweight infants is associated with an independent increased risk of death or neurodevelopment impairment compared with single-gestation infants. However, they overlooked the adverse influences from disasters such as earthquakes. Thus our study provides evidence that further research focused upon twins who have experienced prenatal disasters may be warranted.

There are a number of limitations to the present study. First, the sampling area is only the minor portion of the devastated region of the Wenchuan earthquake, so it might not fully represent the overall conditions. Second, the relatively small sample size of twins and birth defects may have led to inadequate statistical power and warrants caution in interpretation of the data. Last, we did not include a few pregnancies inside the survey area who subsequently moved outside the earthquake area, as data on these cases were generally unavailable. Regardless our data provide new and relevant information on the consequences of a natural disaster on birth outcomes.
